# The Prevalence and Risk Factors of Stress Urinary Incontinence Among Women in Saudi Arabia: A Systematic Review and Meta-Analysis

**DOI:** 10.3390/healthcare12232440

**Published:** 2024-12-04

**Authors:** Mohammed Abdullah Saad Alhamoud, Fatimah Ali Julaih, Hadi Dhafer Hadi Al-Aqil, Naif Abdullah S. Almalki, Faisal Abdullah G. Alharthi, Ahmed Abdullah Alghamdi, Sulaiman Ali K. Alshehri, Ahmad Saeed Alqhtani, Mohammed Abdulrahman Alasiri, Abdullah Sulaiman Alaqil, Bandar Naffaa Alhumaidi, Ariana Saraiva, Conrado Carrascosa, António Raposo

**Affiliations:** 1 Ministry of Health, Abha 63521, Saudi Arabia; mohd2617@gmail.com; 2 College of Medicine, Imam Abdulrahman bin Faisal University, Dammam 31451, Saudi Arabia; fatimah_julaih@outlook.com; 3 College of Medicine, Najran University, Najran 66462, Saudi Arabia; hadi0559317793@gmail.com; 4 Armed Forces Hospital, Dhahran 34641, Saudi Arabia; almalki-naif1992@hotmail.com (N.A.S.A.); f.alharthi1023@gmail.com (F.A.G.A.); 5 Prince Sultan Military College of Health Sciences, Dhahran 31932, Saudi Arabia; ahmedalghamdi1@hotmail.com (A.A.A.); sulaiman-_@hotmail.com (S.A.K.A.); aborakanq009@gmail.com (A.S.A.); tt36@windowslive.com (M.A.A.); 6 King Fahad Military Medical Complex, Dhahran 31932, Saudi Arabia; aboodi09090@gmail.com; 7 Department of Community Health Nursing, College of Nursing, Taibah University, Al Madinah Al Munawwarah 42241, Saudi Arabia; bhumide@taibahu.edu.sa; 8 Research in Veterinary Medicine (I-MVET), Faculty of Veterinary Medicine, Lisbon University Centre, Lusófona University, Campo Grande 376, 1749-024 Lisbon, Portugal; ariana.saraiva@ulusofona.pt; 9 Department of Animal Pathology and Production, Bromatology and Food Technology, Faculty of Veterinary, Universidad de Las Palmas de Gran Canaria, Trasmontaña s/n, 35413 Arucas, Spain; conrado.carrascosa@ulpgc.es; 10 CBIOS (Research Center for Biosciences and Health Technologies), Universidade Lusófona de Humanidades e Tecnologias, Campo Grande 376, 1749-024 Lisbon, Portugal

**Keywords:** meta-analysis, prevalence, risk factors, Saudi Arabia, stress urinary incontinence, women

## Abstract

Background: Stress urinary incontinence (SUI) is a prevalent condition among women in Saudi Arabia, characterized by involuntary urine leakage during physical activities that increase abdominal pressure, such as coughing or sneezing. This systematic review and meta-analysis aimed to evaluate the prevalence of SUI and identify its key risk factors. Methods: A comprehensive search of PubMed, Scopus, and Web of Science was conducted for studies published up to July 2024, following PRISMA 2020 guidelines. Results: Ten observational studies involving 18,245 participants met the inclusion criteria, and study quality was assessed using the Newcastle–Ottawa Scale. A random-effects model was employed for meta-analysis, with subgroup and sensitivity analyses performed to address heterogeneity. The pooled prevalence of SUI was 26% (95% CI: 14–41%, I^2^ = 99%, *p* < 0.001), with rates ranging from 3.3% to 50%. Subgroup analysis showed a prevalence of 17% (95% CI: 1–42%, I^2^ = 99%, *p* < 0.001) in the general population and 33% (95% CI: 19–48%, I^2^ = 99%, *p* < 0.001) in specific groups, such as postpartum women and those with low back pain. Significant risk factors included age, obesity, high parity, and chronic conditions like diabetes. Despite high heterogeneity, sensitivity analyses confirmed the robustness of these findings. Conclusions: The findings underscore the need for public health strategies focused on weight management, pelvic floor rehabilitation, and increased awareness about SUI. Effective preventive measures could significantly reduce the burden of SUI and improve the quality of life for women in Saudi Arabia.

## 1. Introduction

Stress urinary incontinence (SUI) is a condition characterized by the involuntary leakage of urine during activities that increase abdominal pressure, such as laughing, coughing, sneezing, or physical exertion [1]. It is a subtype of urinary incontinence (UI), broadly defined as the involuntary loss of urine. According to the International Continence Society (ICS), SUI occurs when the pelvic floor muscles fail to provide adequate support to the bladder and urethra during pressure-inducing activities, leading to urine leakage [2,3]. This condition is highly prevalent among women, with global estimates suggesting that 29% to 75% experience SUI at some point in their lives [4].

Several factors contribute to the development of SUI, including age, obesity, high parity, and pregnancy-related changes. Childbirth, particularly vaginal delivery, weakens the pelvic floor muscles, increasing susceptibility to the condition [5]. Other contributing factors include a high body mass index (BMI), chronic diseases such as diabetes, and lower levels of physical activity [6,7].

Beyond its physical symptoms, SUI significantly impacts psychological and social well-being. Women with SUI often report reduced quality of life due to embarrassment, social isolation, and limitations in physical and social activities [8]. The stigma associated with incontinence exacerbates these challenges, leading to underreporting and delays in seeking treatment [9]. Additionally, concerns about urine leakage during intimate moments may strain personal relationships and further diminish the quality of life [10].

In Saudi Arabia, the prevalence of SUI is influenced by high rates of obesity, multiparity, and cultural barriers to discussing urinary incontinence [11]. Despite its significant health and social impact, research on SUI among Saudi women remains sparse. Approximately ten studies have explored the prevalence of SUI in Saudi Arabia, targeting various groups such as outpatient clinic patients, community-based samples, first-time mothers, women with low back pain, and the general population [12,13,14,15,16,17,18,19,20,21].

To address this gap, this systematic review and meta-analysis aimed to provide a comprehensive estimate of the prevalence of SUI among Saudi women and identify its key risk factors. By synthesizing data from diverse populations and regions, this study seeks to inform public health strategies to better manage and prevent SUI in Saudi Arabia

## 2. Materials and Methods

### 2.1. Eligibility Criteria

We included observational studies (cross-sectional, cohort, or case-control) that evaluated the prevalence and risk factors of stress urinary incontinence (SUI) among women in Saudi Arabia. Eligible studies were required to report detailed information on the diagnosis of SUI, its prevalence, and the associated risk factors. Studies needed to be published in English and conducted within Saudi Arabia to be considered. Studies focusing on other types of urinary incontinence (UI) without providing a breakdown for SUI were excluded, as were non-observational studies, such as clinical trials, case reports, reviews, and editorials. Subpopulations without broader relevance to the general female population in Saudi Arabia were also excluded.

### 2.2. Search Strategy

A comprehensive search was conducted across PubMed, Scopus, and Web of Science to identify relevant studies, adhering to the PRISMA 2020 guidelines [22]. The search was completed in July 2024 and utilized a combination of Medical Subject Headings (MeSH) terms and free-text keywords, employing Boolean operators (“AND” and “OR”) to maximize inclusivity. For example, the search string used in PubMed was as follows: (“urinary incontinence” OR “UI”) AND “women” AND “Saudi Arabia”. In Scopus, the search included combinations such as TITLE-ABS-KEY (“urinary incontinence” OR “UI”) AND “women” AND “Saudi Arabia”. Similar strategies were implemented across all databases. The search strategies adopted in each database are presented in the Table S1.

The total number of records retrieved from each database was documented and is available as Supplementary Materials. Additionally, the reference lists of the included studies were screened manually to ensure that no relevant studies were missed.

### 2.3. Study Selection

All identified records were imported into EndNote software to remove duplicates. Two independent reviewers screened the titles and abstracts of the studies to exclude irrelevant articles. The full texts of potentially eligible studies were reviewed to ensure that they met the inclusion criteria. Discrepancies between reviewers were resolved through discussion or by consulting a third reviewer. The PRISMA flowchart documents the detailed selection process, including the number of records retrieved, screened, and excluded at each step.

### 2.4. Data Collection

Data were systematically extracted using a predefined data extraction form. Extracted variables included the study type, sample size, participant characteristics (e.g., age, population type), region in Saudi Arabia, prevalence of SUI, diagnostic methods (e.g., self-reported questionnaires, clinical assessments), and identified risk factors (e.g., obesity, parity, diabetes).

### 2.5. Quality Assessment

The quality of the included studies was assessed using the Newcastle–Ottawa Scale (NOS), evaluating three key domains: selection of the study groups, comparability of groups, and ascertainment of outcomes. Studies were scored on a scale of 0 to 9 and categorized as high quality (7–9), moderate quality (4–6), or low quality (below 4). Two independent reviewers conducted the quality assessments, and any discrepancies were resolved through consensus.

### 2.6. Statistical Analysis

Statistical analyses were performed using MetaXL version 5.3 (EpiGear International, Sunrise Beach, Australia). A random-effects model was used to calculate the pooled prevalence of SUI, with 95% confidence intervals (CIs). Heterogeneity among studies was assessed using Higgins’ I^2^ statistic and Cochran’s Q test. Values of I^2^ above 50% were considered indicative of significant heterogeneity. Forest plots were generated to display individual study estimates and overall pooled prevalence. Subgroup analyses were conducted to explore potential sources of heterogeneity, including differences in population types. Sensitivity analyses were performed to assess the impact of outlier studies, using Baujat plots created with Python version 3.9 (Matplotlib version 3.4.3 and Pandas version 1.3.5) Publication bias was evaluated using funnel plots, with further statistical testing if asymmetry was observed.

## 3. Results

### 3.1. Article Screening and Selection

**Figure 1:**
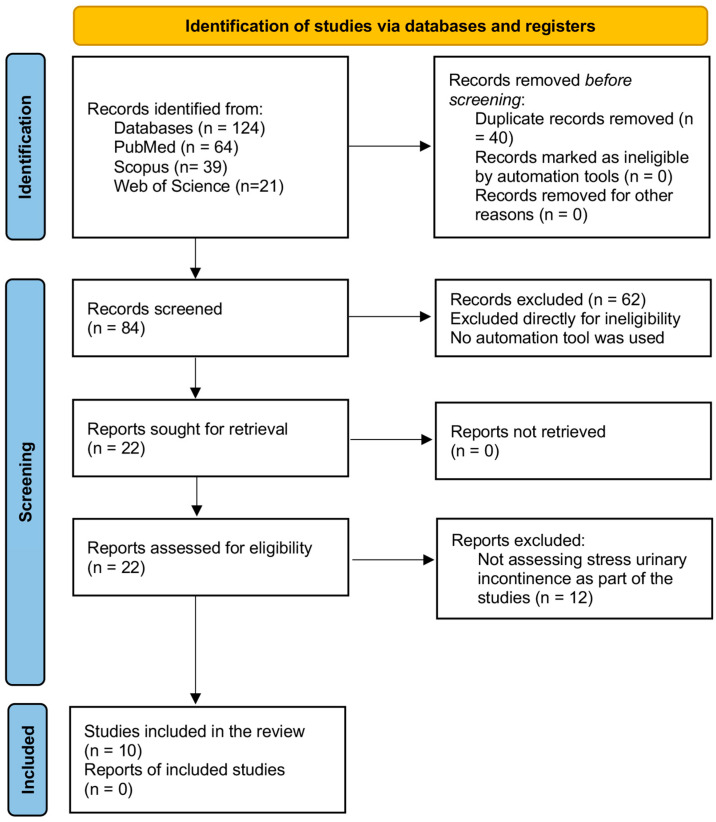
PRISMA flow diagram. PRISMA = the preferred reporting items for systematic reviews and meta-analyses.

A total of 124 publications were identified via database searches (Figure 1). After removing 40 duplicate records, 84 records remained for screening. During this phase, 62 records were excluded due to ineligibility, including non-research articles and off-topic studies. Subsequently, 22 full-text articles were evaluated for eligibility, leading to the exclusion of 12 studies: 7 due to irrelevant populations and 5 because of insufficient data. Ultimately, 10 studies that met the inclusion criteria were included in the qualitative synthesis. These studies were conducted in various regions of Saudi Arabia and assessed the prevalence and risk factors of SUI among women.

### 3.2. Characteristics of the Included Studies

The 10 studies included in this review varied in sample sizes, ranging from 285 to 6600 participants, and targeted different populations, such as women attending primary healthcare centers, the general population, and special groups like women with low back pain (Table 1). The age range of participants across studies spanned from 15 to 72 years. The overall prevalence of SUI varied considerably from 3.3% (Gari et al., 2023) to 50% (Altaweel et al., 2012) [11,14]. The method of diagnosing SUI primarily involved self-reported symptoms through structured questionnaires, except for two studies that used validated diagnostic tools. Several studies identified significant risk factors associated with SUI, including age, obesity, high parity, and chronic health conditions. For example, Al-Badr et al. (2012) reported a prevalence of 36.4%, with significant associations between SUI and factors such as menopause, chronic cough, and constipation [20]. Altaweel et al. (2012), reporting the highest prevalence of 50%, emphasized the strong association between SUI and obesity, diabetes, high parity, and large baby birth weight [14].

### 3.3. Prevalence of Stress Urinary Incontinence

**Figure 2:**
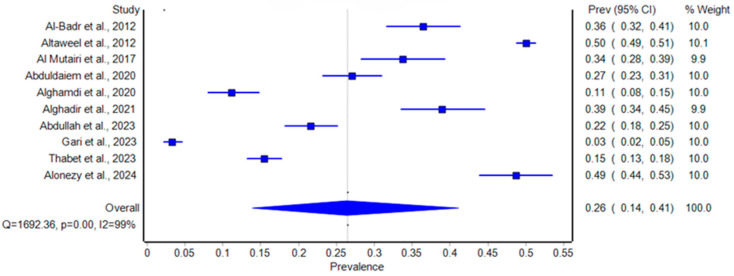
Forest plot of the prevalence of stress urinary incontinence among women in Saudi Arabia [11,12,13,14,15,16,17,18,19,20].

The pooled prevalence of SUI among women in Saudi Arabia was estimated at 26% (95% CI: 14% to 41%) (Figure 2). The prevalence rates across the studies varied widely, from 3.3% to 50%, contributing to significant heterogeneity (I^2^ = 99%, *p* < 0.001). The highest prevalence was observed in the study by Altaweel et al. (2012), while the lowest was found in the study by Gari et al. (2023) [11,14]. This heterogeneity could be attributed to differences in study populations, methods of diagnosis, and regional differences in the prevalence of SUI. The high heterogeneity observed (I^2^ = 99%, *p* < 0.001) indicates variability among studies, suggesting that the results should be interpreted with caution. Potential sources of this heterogeneity include differences in population characteristics, diagnostic methods, and study designs.

**Figure 3:**
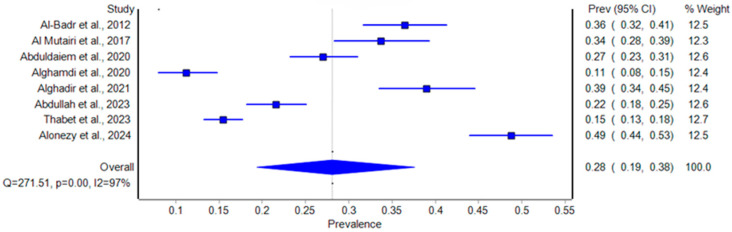
Forest plot of the prevalence of stress urinary incontinence among women in Saudi Arabia (after excluding outliers) [12,13,15,16,17,18,19,20].

A separate meta-analysis of studies that focused specifically on women from the general population reported a pooled prevalence of 17% (95% CI: 1% to 42%) (Figure 3). The individual prevalence rates ranged from 3.3% to 48.6%, with significant heterogeneity remaining (I^2^ = 99%, *p* < 0.001). For instance, Alonezy et al. (2024) reported a prevalence of 48.6%, identifying obesity, age, and number of children as significant contributing factors [16].

**Figure 4:**
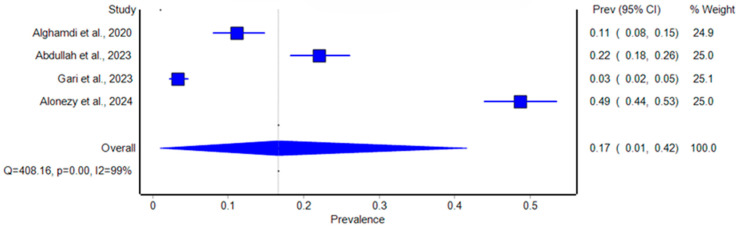
Forest plot of the prevalence of stress urinary incontinence among women from the general population in Saudi Arabia [11,12,15,16].

**Figure 5:**
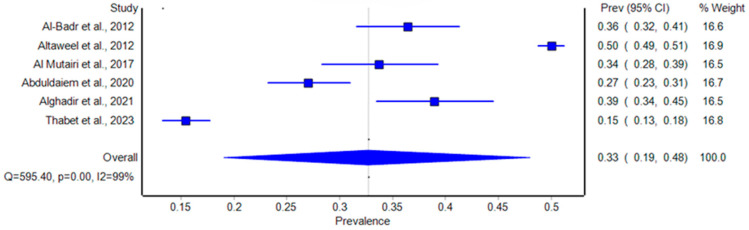
Forest plot of the prevalence of stress urinary incontinence among specific groups (non-general population) of women in Saudi Arabia [13,14,17,18,19,20].

For studies that focused on non-general population women, the pooled prevalence was estimated at 33% (95% CI: 19% to 48%) (Figure 4). Prevalence varied significantly between studies, with Altaweel et al. (2012) reporting the highest rate of 50%, while Thabet et al. (2023) reported a lower rate of 15% [13,14]. The high heterogeneity (I^2^ = 99%, *p* < 0.001) may stem from differences in study design, population characteristics, and diagnostic criteria. Notably, this subgroup includes women with specific conditions, such as those with low back pain or those postpartum, where factors like obesity, parity, and chronic conditions such as diabetes were associated with an increased prevalence of SUI (Figure 5).

### 3.4. Risk Factors for Stress Urinary Incontinence

Several consistent risk factors for SUI were identified across the included studies. Age was a prominent factor, with older women being more prone to SUI. For example, both Al-Badr et al. (2012) and Abduldaiem et al. (2020) reported that women over the age of 35 had a significantly higher risk of developing SUI [19,20]. Similarly, obesity was highlighted as a key risk factor in multiple studies, including Altaweel et al. (2012) and Alghamdi et al. (2020), with these studies showing a strong correlation between high body mass index (BMI) and the prevalence of SUI [12,14]. High parity, or the number of childbirths, was also identified as a significant risk factor. Studies by Al-Badr et al. (2012) and Abduldaiem et al. (2020) reported that having five or more children was strongly associated with SUI [19,20]. Chronic diseases, including hypertension and diabetes, were additional factors that increased the likelihood of SUI, as highlighted in studies by Abduldaiem et al. (2020) and Abdullah et al. (2023) [15,19]. Other contributing factors included menopause, previous pelvic or abdominal surgery, and chronic conditions like low back pain. Alghadir et al. (2021) found a unique association between low back pain and SUI, suggesting that women with core muscle dysfunction, such as those with low back pain, may have an increased risk of SUI [17]. This relationship was further supported by the study’s findings, where 77% of women with low back pain reported urine leakage during coughing or sneezing.

### 3.5. Severity and Impact on the Quality of Life

The severity of SUI varied across the studies, with many women experiencing urine leakage during activities such as coughing, sneezing, or exercising (Table 2). For instance, Alghamdi et al. (2020) reported that 69.3% of obese women experienced urine leakage during coughing or sneezing, while Alghadir et al. (2021) found that 77% of women with low back pain had similar symptoms [12,17]. The studies also explored the impact of SUI on the quality of life. Al-Badr et al. (2012) found that 29.9% of women with SUI reported limitations in social activities, while Thabet et al. (2023) found that SUI had a profound impact on both mental health and social life, with 39.64% of women reporting adverse effects on mental health [13,20]. Alonezy et al. (2024) reported similar findings [16].

### 3.6. Study Biasness and Sensitivity Identification

**Figure 6:**
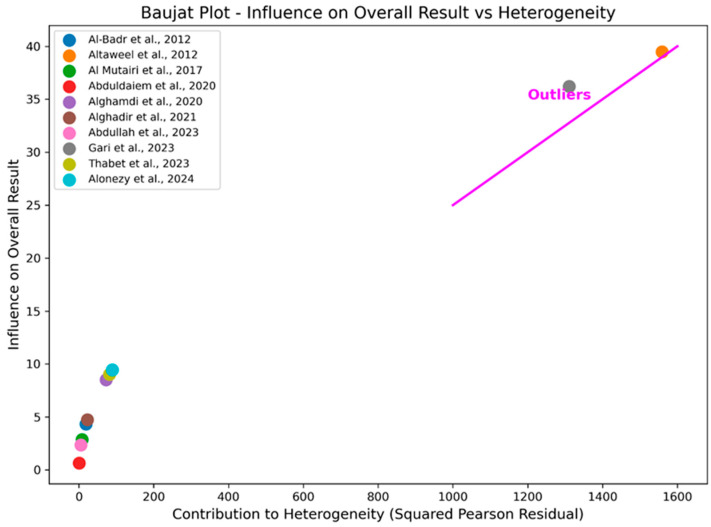
Baujat plot highlighting study contributions to heterogeneity and influence on the overall SUI prevalence estimates [11,12,13,14,15,16,17,18,19,20].

The Baujat plot (Figure 6) identified Altaweel et al. (2012) and Gari et al. (2023) as significant outliers, contributing heavily to heterogeneity and strongly influencing the overall results [11,14]. Altaweel et al. (2012) had a prevalence of 50% with a confidence interval (CI) of 0.49 to 0.51 among 6600 participants, while Gari et al. (2023) had a much lower prevalence of 3.3% (CI: 0.02 to 0.05) among 842 participants [11,14]. Both studies were determined to be potential sources of bias due to their positions on the top–right of the Baujat plot. To assess the impact of these outliers on the pooled estimate, we generated a forest plot after excluding them (Figure 3). The initial pooled prevalence was 26% (CI: 0.14 to 0.41) with high heterogeneity (I^2^ = 99%, Q = 1692.36, *p* < 0.001). After excluding Altaweel et al. (2012) and Gari et al. (2023), the pooled prevalence increased slightly to 28% (CI: 0.19 to 0.38) with a reduced heterogeneity (I^2^ = 97%, Q = 271.51, *p* < 0.001) [11,14]. Despite the robustness of pooled estimates after sensitivity analysis, the high heterogeneity (I^2^ = 97%) underscores the need for the cautious interpretation of these results. This sensitivity analysis demonstrates that while the exclusion of these outliers decreased the heterogeneity, the overall prevalence remained relatively stable, confirming the robustness of the pooled estimates.

### 3.7. Quality Assessment of the Included Studies

Table 3 summarizes the quality assessment of the included studies, which indicated that four studies were classified as high quality (Altaweel et al., 2012; Alghadir et al., 2021; Gari et al., 2023; Thabet et al., 2023) and the remaining six studies were classified as moderate quality [11,13,14,17]. The studies shared common limitations, such as the lack of rigor in the assessment of outcomes and the selection of participants.

## 4. Discussion

This systematic review and meta-analysis aimed to determine the prevalence of SUI and identify its risk factors in women in Saudi Arabia. The results indicated a significant variation in the prevalence of SUI, ranging from 3.3% to 50%, depending on the study population and methodology employed. The pooled prevalence was estimated at 26%, but substantial heterogeneity was noted, reflecting differences in study populations, diagnostic methods, and regional variations. Most studies relied on self-reported symptoms, which may lead to underreporting or overestimation due to varying levels of comfort in discussing sensitive issues [16,20]. In contrast, studies utilizing clinical evaluations or validated diagnostic tools provided more reliable prevalence estimates. Subgroup analyses revealed that the prevalence was lower in the general female population (17%) but higher among specific groups such as postpartum women or those with chronic health conditions (33%). These findings highlight the importance of considering study context and methodology when interpreting prevalence rates.

Interestingly, studies focusing on the general female population in Saudi Arabia reported a lower pooled prevalence of 17%, while those examining specific subgroups, such as postpartum women or those with chronic health conditions, showed a higher pooled prevalence of 33%. This trend suggests that certain groups are at an elevated risk for SUI, aligning with existing literature that identifies pregnancy, childbirth, obesity, and chronic conditions like diabetes as significant contributors to SUI development.

When comparing these findings with global data, it becomes evident that the prevalence of SUI in Saudi Arabia falls within the global range but shows substantial regional variability. A systematic review by Saba et al. (2022) indicated that global SUI prevalence can reach up to 49%, with marked differences based on demographic factors and geographical regions [23]. For instance, studies in some European countries report a prevalence of around 35% for urinary incontinence, including SUI [24]. Higher rates have been observed in certain cohorts; Brown et al. found a 55% prevalence, while Black et al. reported rates as high as 62%, further highlighting the discrepancies attributable to study design, population characteristics, and differing definitions of urinary incontinence [24].

The prevalence of SUI observed in Saudi Arabia aligns with global trends but reflects notable regional variability. A systematic review by Saba et al. (2022) reported that global SUI prevalence can reach 49%, with significant differences based on demographic and geographical factors [23]. For example, studies in Europe report urinary incontinence rates of approximately 35%, while some Asian countries, including Indonesia and India, have shown higher prevalence rates among postpartum or multigravid women, ranging from 40% to 55% [25,26]. These variations are influenced by the differences in population characteristics, healthcare access, and cultural factors. Comparisons with global data reinforce the importance of addressing SUI within a region-specific context, particularly in populations with unique risk profiles. Similarly, in Brazil, mixed urinary incontinence affected 36.2% of women, with stress incontinence contributing to 24.2% of cases [27].

Our results identified several consistent risk factors for SUI in women in Saudi Arabia, including age, obesity, high parity, and chronic health disorders such as diabetes. These findings align with global research, underscoring the universal nature of these risk factors. In our review, age was a prominent factor, with studies indicating a higher prevalence of SUI among women over the age of 35, particularly after 40. This is consistent with global data, which shows that aging is a key contributor to pelvic floor weakening, increasing the likelihood of SUI [28]. Similarly, international studies from Europe and North America indicate that older women have an elevated risk due to diminished pelvic muscle tone, with prevalence rates varying between 25% and 55%, depending upon the population examined [29].

Obesity was another major risk factor in our review, particularly in a population like Saudi Arabia, where obesity rates are high. Our findings, showing a strong correlation between high BMI and SUI prevalence, are in line with global evidence, which links increased intra-abdominal pressure due to excess weight with higher rates of incontinence [28]. International studies have also indicated that for each 1 kg/m^2^ rise in BMI, the likelihood of SUI escalates by 7%, underscoring the worldwide significance of obesity as a modifiable risk factor [29].

High parity also emerged as a significant factor, with women who had five or more childbirths showing a higher risk for SUI. This mirrors global trends, where studies consistently report that multiple pregnancies and vaginal deliveries contribute to pelvic floor damage, increasing SUI prevalence [24,30]. For instance, research from Brazil and India supports our findings, showing a strong association between high parity and SUI, with prevalence rates as high as 55% in women with multiple pregnancies [23].

Chronic health conditions such as diabetes also played a role in our study, reinforcing global evidence that metabolic disorders are key contributors to SUI. International research has similarly found that diabetes and other chronic conditions disrupt urinary control mechanisms, exacerbating the risk of incontinence [31]. This further highlights the multifactorial nature of SUI across different populations.

An interesting and unique finding from the Saudi context was the association between SUI and low back pain, as identified by Alghadir et al. (2021) [17]. This study suggested that women with core muscle dysfunction, such as those with chronic back pain, might have weaker pelvic floor muscles, leading to a higher SUI risk. This relationship underscores the interconnected nature of musculoskeletal health and pelvic floor function, which may require further exploration in future research [32].

The impact of SUI on the quality of life was another key theme across studies. Many women reported avoiding social activities, physical exertion, and intimate relationships due to fear of leakage. The psychological burden of SUI, including anxiety, depression, and social isolation, was highlighted in several studies [15,16,19]. For instance, Al-Badr et al. (2012) found that nearly 30% of women with SUI experienced limitations in social engagements, while Alghadir et al. (2021) emphasized the negative correlation between SUI and musculoskeletal health [17,20]. Table 2 further illustrates the severity of SUI symptoms and their impact on daily life, showing that women with SUI frequently experience significant disruptions in their mental and physical well-being. Addressing these quality-of-life issues is critical for improving outcomes and ensuring that women receive appropriate support and treatment.

### Limitations of the Study

This systematic review and meta-analysis provide valuable insights; however, several limitations must be acknowledged. Firstly, the high heterogeneity among the included studies complicates the interpretation of pooled prevalence estimates. The observed variability likely stems from differences in study design, diagnostic criteria, and the demographic and clinical characteristics of the populations studied. Secondly, the reliance on self-reported data in many of the studies introduces the potential for reporting bias, which could either overestimate or underestimate the actual prevalence of SUI. Thirdly, the absence of standardized diagnostic tools across studies further limits the reliability and comparability of findings. Lastly, many of the included studies focused on specific subpopulations, such as postpartum women or those with low back pain, which restricts the generalizability of the results to the broader female population in Saudi Arabia. Addressing these limitations in future research is essential to provide a more comprehensive understanding of SUI prevalence and its risk factors.

## 5. Conclusions

This systematic review and meta-analysis highlight the substantial prevalence of stress urinary incontinence (SUI) among women in Saudi Arabia, with a pooled prevalence of 26%, though significant variability was observed across studies. Age, obesity, high parity, and chronic diseases such as diabetes were identified as significant risk factors for the development of SUI. The condition has a profound impact on women’s quality of life, contributing to physical discomfort and psychological distress. Additionally, cultural stigmas surrounding incontinence often result in underreporting and delayed treatment, further exacerbating the burden of SUI. To address these issues, public health initiatives should prioritize increasing awareness of SUI, promoting early diagnosis, and addressing modifiable risk factors, such as obesity and childbirth-related pelvic trauma, through targeted educational and intervention programs. Future research should explore culturally sensitive approaches to improve the management and treatment of SUI in Saudi Arabia. By addressing both the physical and psychosocial challenges associated with SUI, healthcare providers can better support affected women and enhance their overall well-being.

## Figures and Tables

**Table 1:** Characteristics of the included studies.

Authors	Study Design	Sample Size	Age Range (Years)	Population	Location (Region in Saudi Arabia)	Number of SUI Cases	Prevalence of Stress Urinary Incontinence (SUI) (%)	Method of Diagnosing	Key Findings
Gari et al. (2023) [11]	Cross-sectional	842	20 and older	Saudi women from the general population	Various regions of Saudi Arabia	28	3.30%	Self-reported symptoms through a structured questionnaire	SUI was associated with older age, widowhood, family history of SUI, and pregnancy history. Family history of SUI increased the odds of SUI by 19.68 times.
Alghamdi et al. (2020) [12]	Cross-sectional	500	18–50	Adult women recruited from the community	Eastern Saudi Arabia	135	27%	Self-reported symptoms through a structured questionnaire	Obesity and multiparity were major contributing factors to the development of SUI.
Thabet et al. (2023) [13]	Cross-sectional	1005	18–70	Saudi women from the western region attending a maternity and children’s hospital	Western Saudi Arabia	155	15.40%	The Arabic version of the Questionnaire for Urinary Incontinence Diagnosis (QUID)	SUI is more prevalent in women over 35, those with high BMI, and multiparous women. A significant correlation was observed with cesarean section and vaginal surgery.
Altaweel et al. (2012) [14]	Cross-sectional	6600	20 and older	Women attending outpatient clinics in Riyadh	Riyadh, Saudi Arabia	3300	50%	The Arabic version of the short form of the Urogenital Distress Inventory (UDI-6) questionnaire	Obesity, diabetes, high parity, and large baby birth weight were strongly associated with SUI.
Abdullah et al. (2023) [15]	Cross-sectional	543 (108 women admitted to maternity and children’s hospital and 435 women from the general population)	18–59	Admitted women and women from the general population	Al-Kharj, Saudi Arabia	96 (general), 21 (admitted)	17.7% (general population), 19.4% (admitted women)	Self-reported symptoms through a structured questionnaire	Advanced age, multiparity, obesity, and chronic diseases were identified as significant risk factors for SUI.
Alonezy et al. (2024) [16]	Cross-sectional	421	18–60	Adult women in the general population	Al Medina Al Munawara	205	48.60%	Self-reported symptoms through a structured questionnaire	Significant associations with age, number of children, obesity, and previous abdominal or pelvic surgery. Coughing and sneezing were the most common triggers of SUI.
Alghadir et al. (2021) [17]	Case-Control	303 (143 in LBP and 160 in control group)	18–45	Married women with low back pain (LBP) and healthy controls	Riyadh, Saudi Arabia	86 (LBP group), 32 (Control group)	60% in LBP group, 20% in control group	Self-reported symptoms through a structured questionnaire	Core muscle endurance showed a stronger negative correlation with SUI in women with LBP than in healthy controls.
Al Mutairi et al. (2017) [18]	Cross-sectional	285	15–40	Primiparous women (first-time mothers) within one year postpartum	Riyadh, Saudi Arabia	96	33.70%	Self-reported symptoms through a structured questionnaire	Significant relationship found between vaginal laceration, high BMI, and SUI after childbirth.
Abduldaiem et al. (2020) [19]	Cross-sectional	340	18–72	Adult women attending primary health care centers	Riyadh, Saudi Arabia	38	11.20%	Self-reported symptoms through a structured questionnaire	Advanced age, high parity (>5), menopause, obesity, and hypertension were significant risk factors for SUI. Medical advice was sought by only 6.5% of the women with UI.
Al-Badr et al. (2012) [20]	Cross-sectional	379	15–71	Adult women visiting primary healthcare centers	Jeddah, Saudi Arabia	138	36.40%	Self-reported symptoms through a structured questionnaire	Age, parity > 5, menopause, chronic cough, and constipation were significant risk factors for SUI.

SUI: stress urinary incontinence, LBP: low back pain, BMI: body mass index, UI: urinary incontinence.

**Table 2:** Symptoms and severity of urinary incontinence across studies.

Study	Urine Leakage During Coughing or Sneezing	Urine Leakage During Exercise or Bending	Urgent Need to Urinate Before Reaching a Toilet	Severe Stress Incontinence	Impact on Social Life
Gari et al., 2023 [11]	18.10%	5.10%	12%	Not mentioned	4.3% reported severe impact on quality of life.
Alghamdi et al., 2020 [12]	69.3% of obese women	46.39%	72.54%	Not mentioned	12.56% of women with UI symptoms sought medical advice.
Thabet et al., 2023 [13]	55.60%	16.70%	46.50%	27.60%	23.4% affected social life, 39.64% affected mental health.
Altaweel et al., 2012 [14]	50%	Not mentioned	28%	Not mentioned	Less than 10% reported a significant effect on HRQL.
Abdullah et al., 2023 [15]	17.7% (general), 19.4% (admitted)	Not mentioned	12.6% (general), 14.8% (admitted)	Not mentioned	Only 6.7% of the general population and 11.1% of admitted patients sought medical advice.
Alonezy et al., 2024 [16]	48.60%	10.10%	34.50%	3.30%	UI significantly impacted the social and psychological aspects of life.
Alghadir et al., 2021 [17]	77% during coughing or sneezing in LBP group	30% during exercise or heavy lifting	27% moving to the bathroom	Not specifically mentioned	SUI was significantly associated with low back pain and affected quality of life.
Al Mutairi et al., 2017 [18]	33.70%	Not mentioned	14.20%	Not mentioned	High BMI and vaginal laceration affected social life.
Abduldaiem et al., 2020 [19]	11.20%	Not mentioned	Not mentioned	Not mentioned	6.5% sought medical advice due to impact on life.
Al-Badr et al., 2012 [20]	36.40%	Not mentioned	27.40%	17.20%	29.9% had limitations in social activities.

UI: urinary incontinence, SUI: stress urinary incontinence, LBP: low back pain, BMI: body mass index, HRQL: health-related quality of life.

**Table 3:** Quality assessment of the included studies.

Study	Participant Selection (4 Points)	Group Comparability (2 Points)	Assessment of Exposure/Outcome (3 Points)	Cumulative Score (9 Points)	Quality Rating
Gari et al., 2023 [11]	4	2	2	8	High Quality
Alghamdi et al., 2020 [12]	2	1	1	4	Moderate Quality
Thabet et al., 2023 [13]	4	2	2	8	High Quality
Altaweel et al., 2012 [14]	3	1	2	6	Moderate Quality
Abdullah et al., 2023 [15]	4	2	2	8	High Quality
Alonezy et al., 2024 [16]	3	1	2	6	Moderate Quality
Alghadir et al., 2021 [17]	3	1	2	6	Moderate Quality
Al Mutairi et al., 2017 [18]	3	1	2	6	Moderate Quality
Abduldaiem et al., 2020 [19]	4	1	2	7	High Quality
Al-Badr et al., 2012 [20]	4	2	2	8	High Quality

## Data Availability

Data sharing is not applicable. No new data were created or analyzed in this study.

## Supplementary Materials

The following supporting information can be downloaded at: https://www.mdpi.com/article/10.3390/healthcare12232440/s1, Table S1: The search strategies adopted in each database.
