# Supplementary material for: The Prevalence and Risk Factors of Stress Urinary Incontinence Among Women in Saudi Arabia: A Systematic Review and Meta-Analysis
Source: Healthcare (Basel). 2024 Dec 4;12(23):2440. doi: 10.3390/healthcare12232440 (PMC11640814; doi:10.3390/healthcare12232440)
Supplement: Supplementary file 1 [file healthcare-12-02440-s001.zip › healthcare-3318807-supplementary.pdf]

Supplementary Materials

Table S1 The search strategies adopted in each database

PubMed	Search Query: (“urinary incontinence” OR “UI”) AND “women” AND “Saudi Arabia”<br>Filters Applied: English, observational studies<br>Records Retrieved: 64<br>Search Date: July 2024
Scopus	Search Query: TITLE-ABS-KEY (“urinary incontinence” OR “UI”) AND “women” AND “Saudi Arabia”<br>Filters Applied: English, observational studies<br>Records Retrieved: 39<br>Search Date: July 2024
Web of Science	Search Query: ("urinary incontinence" OR "UI") AND "women" AND "Saudi Arabia"<br>Filters Applied: English, observational studies<br>Records Retrieved: 21<br>Search Date: July 2024
Study Selection Summary	Total Records Identified: 124<br>Records After Duplicates Removed: 84<br>Records Screened: 84<br>Records Excluded: 62<br>Reports Sought for Retrieval: 22<br>Reports Assessed for Eligibility: 22<br>Reports Excluded (Not assessing SUI): 12<br>Total Studies Included in Review: 10
